# Ordering and Reverse Ordering Mechanisms of Triblock Copolymers in the Presence of Solvent

**DOI:** 10.3390/ijms10030805

**Published:** 2009-02-27

**Authors:** Panagiotis Maniadis, Kim Ø. Rasmussen, Russell B. Thompson, Edward M. Kober

**Affiliations:** 1 Theoretical Division and Center for Nonlinear Studies, Los Alamos National Laboratory, Los Alamos, New Mexico, 87545, USA; 2 Department of Physics & Astronomy, University of Waterloo, Waterloo, Ontario, N2L 3G1, Canada

**Keywords:** Triblock copolymers, self-assembly, self-consistent field theory

## Abstract

Self-consistent field theory is used to study the self-assembly of a triblock copolymer melt. Two different external factors (temperature and solvent) are shown to affect the self-assembly. Either one or two-step self-assembly can be found as a function of temperature in the case of a neat triblock melt, or as a function of increasing solvent content (for non-selective solvents) in the case of a triblock-solvent mixture. For selective solvents, it is shown that increasing the solvent content leads to more complicated self-assembly mechanisms, including a reversed transition where order is found to increase instead of decreasing as expected, and re-entrant behavior where order is found to increase at first, and then decrease to a previous state of disorder.

## 1. Introduction

Triblock copolymers are linear polymers composed of three separate regions, or “blocks”, of monomers. Triblocks with three different chemical blocks are usually labeled as *ABC* triblocks. These molecules are much less studied than the less complicated diblock copolymers, because the addition of a third chemically distinct block greatly complicates the microphase separation properties of these self-assembling molecules. It is this increased morphological richness however that makes triblocks appealing, as they can form structures inaccessible to diblocks. It is not just a question of which structures will self-assemble, but also the manner in which the self-assembly takes place. Diblocks can only be in an ordered state or a disordered state, whereas triblock copolymers are the simplest possible copolymer than can be also in a “semi-ordered” state, where two of the blocks are mixed while the third alone is segregated. Triblock copolymers can therefore go from a completely disordered state to a completely ordered state through a multi-step process that holds promise for technological applications if it can be sufficiently understood and predicted.

Experimental studies of complex ordering in triblock systems were undertaken by Yamauchi [1, 2] who examined polyisoprene-*block*-poly (D_8_-styrene)-*block*-poly(vinyl methyl ether) (PI*-b-*PDS*-b-*PVME) triblock terpolymers and found that ordering may proceed via a two-phase mechanism. These authors [2] demonstrated that the asymmetry in the temperature dependence of the miscibility of these three components may be used to differentiate between one-step and two-step segregation. Also, it was shown [1] that segregation of one species from the other two occurred first, followed by complete microphase separation between all three chemical species as the solvent content was increased. Since solvent generally dilutes monomer interactions and thus drives the system toward increased miscibility this at first sight appears counter intuitive, but we shall here show that strongly selective solvent indeed may produce the observed effect. A preliminary theoretical investigation [3] used self-consistent field theory (SCFT) to clarify the self-assembly in terms of the energies and entropies of the system. This version of the theory predicted both the one-step (transition from fully disordered to fully ordered) and two-step (fully disordered to semi-ordered to fully ordered) self-assembly possibilities observed experimentally. However, it did so through varying the system parameters in a way that does not closely correspond to approaches practical in applications, or that have been taken in the experiments [1, 2].

Here, we build on the previous theoretical results [3] and show, using SCFT, that multi-step self-assembly in triblock systems is predicted to be not only still present under more realistic circumstances, but that the process is predicted to be much richer than found in either the previous idealized case [3] or in experiments to date [1, 2]. We use SCFT to examine a triblock copolymer melt with more realistic temperature segregation variations, and triblock systems involving both non-selective and selective solvents. Self-assembly mechanisms that arise both through varying temperature and through varying non-selective solvent volume fraction reproduce experimental, and previous theoretical findings of one and two-step ordering. Further though, changing the volume fraction of selective solvent is predicted to induce much more exotic ordering in which the fully and semi-ordered phases alternate as volume fraction of solvent is increased. The system thus shows re-entrant behavior in the self-assembly process.

## 2. Self-Consistent Field Theory

Equilibrium morphologies for a neat, incompressible melt of monodisperse triblock copolymers can be calculated from the free energy functional given in ref. [3]. The parameters specifying the nature of triblock melt are ${\text{X}}_{AB}$*N*, ${\text{X}}_{BC}$*N*, ${\text{X}}_{AC}$*N*, *f_A_*, *f_C_*, *a_A_*/*a_C_* and *a_B_*/*a_C_*. These are, respectively, the segregation parameters between *A* and *B* segments, *B* and *C* segments, *A* and *C* segments, the volume fraction of *A* segments, the volume fraction of *C* segments, the ratio of the *A* statistical segment length to the *C* statistical segment length and the ratio of the *B* statistical segment length to the *C* statistical segment length. The segregation energies are standardly defined in terms of X*N*, which is the product of the Flory-Huggins parameters ${\text{X}}_{ij}$ and the total number of segments *N* in a single triblock molecule, based on a reference segment volume $\rho_{0}^{-1}$, assumed to be the same for all interactions. For the volume fractions, only *f_A_* and *f_C_* need to be specified since through incompressibility *f_B_* is not independent. For the current work, we will simplify the system further by restricting our attention to cases where *f_A_* *= f_C_* ≡ *f_A/C_* = 0.25 (*f_B_* = 1 − *f_A_* − *f_C_* = 0.5), in keeping with the previous work [3]. Similarly, we will parallel that work by assuming all the statistical segment lengths are equal, so that *a_A_* = *a_B_* = *a_C_* ≡ *a*. These restrictions will impose certain symmetries on the system, but should still allow the predominant structures and mechanisms to emerge as previously demonstrated for diblock systems [4]. In our previous work however, we also assumed ${\text{X}}_{AB}$*N* = ${\text{X}}_{BC}$*N* = 50, but more general values will be considered here. In addition, we will also allow for the presence of solvent.

In order to introduce the effect of the solvent in the SCFT, we need to introduce more variables [5]. The first is the volume fraction of polymer, *φ*, with the volume fraction of the solvent being *φ_s_* *=* 1 − *φ*. The second is the molecular volume ratio *α,* which is the ratio of the volume of a solvent molecule to the volume of a polymer molecule. This will be held fixed (*α =* 0.01) in this work. The third set is the interaction of the solvent with the polymer, which is determined by the three parameters ${\text{X}}_{AS}$*N,* ${\text{X}}_{BS}$*N* and ${\text{X}}_{CS}$*N*. The modified SCFT equations then are

(1) $$\frac{\partial q\left(\text{r},s\right)}{\partial s}\;\;=\;\;R_{g}^{2}\nabla^{2}q\left(\text{r},s\right)\;\;-\omega \left(\text{r}\right)\;q\left(r,s\right)$$

with the initial condition *q*(*r,* 0) = 1. *s* is the normalized contour length (0 ≤ *s* ≤ 1) of a polymer chain, and the field *ω*(**r**) is equal to

(2) $$\omega \left(\text{r}\right)=\{\begin{array}{ll} \omega_{A}\left(\text{r}\right), & 0\leq s<f_{A},\\ \omega_{B}\left(\text{r}\right), & f_{A}\leq s<f_{A}+f_{B},\\ \omega_{C}\left(\text{r}\right), & f_{A}+f_{B}\leq s\leq 1. \end{array}$$

Since the two ends of the polymer chain are not equivalent, a propagator *q*^†^ (**r***, s*) is also defined by the equation

(3) $$-\frac{\partial q^{†}\left(\text{r},s\right)}{\partial s}=R_{g}^{2}\nabla^{2}q^{†}\left(\text{r},s\right)-\omega \left(\text{r}\right)q^{†}\left(\text{r},s\right).$$

It is possible to solve these diffusion equations self consistently, together with the equations that connect the densities with the chemical potential fields *ω*(**r**) and the Lagrange multiplier *ξ*(**r**) (which is introduced to force incompressibility)

(4) $$\omega_{i}\left(\text{r}\right)\;\;=\;\;N\sum_{j\neq i}{\text{X}}_{ij}\varphi_{j}\left(\text{r}\right)\;\;-\xi \left(\text{r}\right),$$

and the incompressibility condition

(5) $$\sum_{j}\varphi_{j}\left(\text{r}\right)\;=1$$

where *j = A, B, C,* and *S.* The single chain partition function and the monomer densities are then

(6) Q  = ∫dr  q(r,1)

(7) $$\varphi_{A}\left(\text{r}\right)\;\;=\;\varphi \frac{V}{Q}\int_{0}^{f_{A}}ds\;\;q\left(\text{r},s\right)\;\;q^{†}\left(\text{r},s\right)$$

(8) $$\varphi_{B}\left(\text{r}\right)\;\;=\;\varphi \frac{V}{Q}\int_{f_{A}}^{f_{A}+f_{B}}ds\;\;q\left(\text{r},s\right)\;\;q^{†}\left(\text{r},s\right)$$

(9) $$\varphi_{C}\left(\text{r}\right)\;\;=\;\;\varphi \frac{V}{Q}\int_{f_{A}+f_{B}}^{1}ds\;\;q\left(\text{r},s\right)\;q^{†}\left(\text{r},s\right).$$

The solvent partition function and density are

(10) $$Q_{S}\;\;=\;\int dr\;\text{exp}\left\{-\alpha \omega_{S}\left(\text{r}\right)\right\}$$

(11) $$\varphi_{S}\left(\text{r}\right)\;\;=\;\left(1-\varphi \right)\frac{V}{Q_{S}}\text{exp}\left\{-\alpha \;\omega_{S}\left(\text{r}\right)\right\}.$$

The numerical procedure employed to obtain the solution of these equations is described elsewhere [6,7]. Once the solution is obtained, the free energy *F* is given by

(12) $$\begin{array}{ll} \frac{F}{n\;k_{B}T}= & -\varphi \text{ln}\left[\frac{Q}{V\varphi }\right]-\frac{1-\varphi }{\alpha }\text{ln}\left[\frac{Q_{s}\alpha }{V\left(1-\varphi \right)}\right]\\ & +\;\;\frac{1}{V}\sum_{i,j,i\neq j}N{\text{X}}_{ij}\int dr\;\;\varphi_{i}\left(\text{r}\right)\;\varphi_{j}\left(\text{r}\right)\\ & -\;\;\frac{1}{V}\sum_{j}\int d\text{r}\;\omega_{j}\left(\text{r}\right)\;\;\;\varphi_{j}\left(\text{r}\right). \end{array}$$

The free energy curvature must be checked to ensure that *d*^2^*F/dφ*^2^ *>* 0 for all *φ*; the mixture will macrophase separate if the curvature is negative [8]. In the cases we will examine *d*^2^*F/dφ*^2^ is always positive so that macrophase separation is absent. Overall we have an eight dimensional parameter space: ${\text{X}}_{AB}$*N*, ${\text{X}}_{BC}$*N*, ${\text{X}}_{AC}$*N*, ${\text{X}}_{AS}$*N*, ${\text{X}}_{BS}$*N*, ${\text{X}}_{CS}$*N*, *f_a/c_*, *φ*. Obviously it is difficult to explore the whole space, so we will choose representative points that illustrate the wide variety of self-assembly mechanisms available to the triblock-solvent system. In particular, we wish to explicitly examine temperature dependence, and the effects of both non-selective and selective solvents in order to more closely parallel the experimental methods of Yamauchi *et al.* [1, 2]

## 3. Results and Discussion

### 3.1. Temperature Dependence

**Figure 1.**
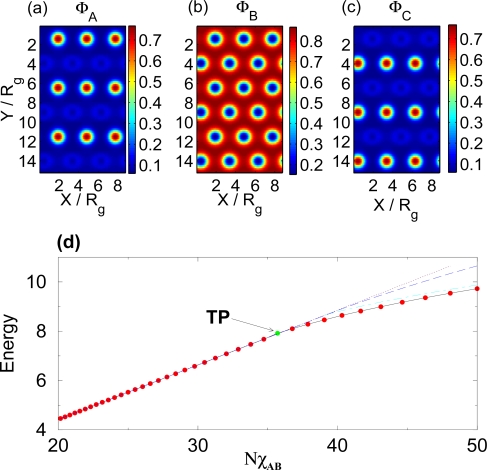
(a), (b) and (c) show spatial monomer densities of the A, B, and C components of the polymer, respectively, for ${\text{X}}_{AB}$*N* = ${\text{X}}_{AC}$*N* = ${\text{X}}_{BC}$*N* = 36 and (d) shows (solid line) the free energy of the melt vs. the Flory-Huggins parameter ${\text{X}}_{AB}$*N* = ${\text{X}}_{BC}$*N* = ${\text{X}}_{AC}$*N*. The point marked TP signifies the one-step order-disorder transition point. Also shown are the free energies of the disordered phase (short-dashed line), the lamellar phase (short-dashed/long-dashed line) and the hexagonal phase in which A and C components mix (long-dashed line).

In Ref. [3], it was shown that one or two-step self-assembly is possible in the triblock system by holding ${\text{X}}_{AB}$*N* *=* ${\text{X}}_{BC}$*N* fixed, and varying ${\text{X}}_{AC}$*N*. Although this shows in principle the possibility of engineering one or two-step self-assembly, it is not amenable to experiment nor is it the approach used by Yamauchi *et al.* [1,2]. Here, we utilize the generally accepted relationship between X (or in our case, X*N*) and temperature [9, 10]

(13) $$\text{X}N\;\;=\;\frac{A}{T}+B$$

where *A* and *B* are constants related to the chemical interactions between species. Ignoring solvent (setting *φ* = 1), we drop *B* for qualitative analysis and we vary all *A’s* equally with temperature such that ${\text{X}}_{AB}$*N* = ${\text{X}}_{BC}$*N* = ${\text{X}}_{AC}$*N* ∝ *T*^−1^. This results in one-step self-assembly as shown in Figure 1. For ${\text{X}}_{AB}$*N* = ${\text{X}}_{BC}$*N* = ${\text{X}}_{AC}$*N* ≲ 35.5 self-assembly is absent and the melt remains in a disordered state. At ${\text{X}}_{AB}$*N* = ${\text{X}}_{BC}$*N* = ${\text{X}}_{AC}$*N* ≃ 35.5 (marked as TP in Figure 1(d)) the system undergoes a one-step phase transition when all three components phase segregate into separate spatial domains as illustrated in Figure 1. The observed one-step self-assembly is therefore a result of the symmetry of the interaction between different components. Figure 1(d) further compares the lowest free energy with the free energies for the morphologies that are close by in the phase diagram. The circles show the minimum free energy, which for ${\text{X}}_{AB}$*N* < 35.5 is the disordered phase where the free energy follows the expected linear dependence of ${\text{X}}_{AB}$*N*. Above TP ( ${\text{X}}_{AB}$*N* > 35.5) the lowest free energy is realized by the hexagonal morphology illustrated in Figure 1(a–c). The energy of the alternative hexagonal phase where the A and C phase mix (see below) is shown by the long-dashed line.

**Figure 2.**
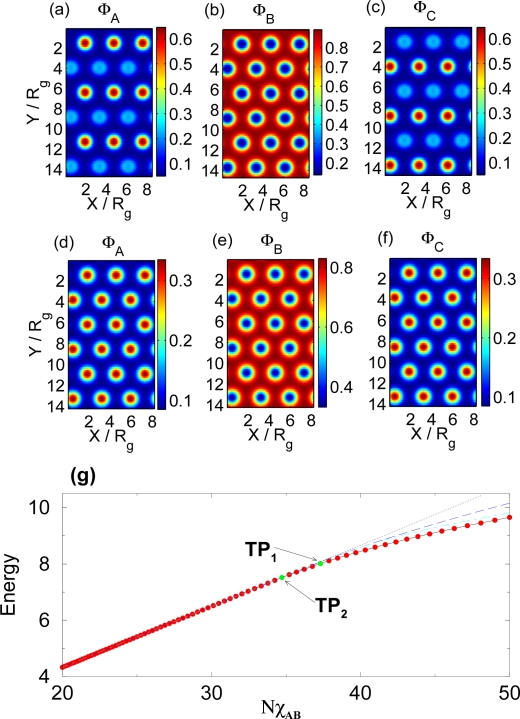
(a), (b) and (c) show spatial monomer densities of the A, B, and C components of the polymer, respectively, after the secondary transition (see text for details) for ${\text{X}}_{AB}$*N* = ${\text{X}}_{BC}$*N* = 37.3 and ${\text{X}}_{AC}$*N* = 28.3. (d), (e) and (f) show spatial monomer densities in between the primary and secondary transition for ${\text{X}}_{AB}$*N* = ${\text{X}}_{BC}$*N* = 34.7 and ${\text{X}}_{AC}$*N* = 26.3. (g) shows the free energy of the melt as a function of the Flory-Huggins parameter ${\text{X}}_{AB}$*N* = ${\text{X}}_{BC}$*N* = $\frac{3}{4}{\text{X}}_{AC}$*N*. The two transition points (*TP*_1_) and (*TP*_2_) are also shown. Also shown are the free energies of the disordered phase (short-dashed line), the lamellar phase (short-dashed/long-dashed line) and the hexagonal phase in which A and C components mix (long-dashed line).

If this symmetry in the interaction is now broken for any reason, then the transition will take place in two separate steps. There are many possible ways of lifting this symmetry as there are no known constraints on the values of *A* and *B* in Equation (13). One simple way to break the symmetry in the interaction that we will examine here is by choosing *A_AB_* = *A_BC_* ≠ *A_AC_* in Equation (13). This corresponds again to allowing all ${\text{X}}_{AB}$*N*, ${\text{X}}_{BC}$*N* and ${\text{X}}_{AC}$*N* to have temperature dependent and independent terms, but with ${\text{X}}_{AC}$*N* having a different temperature dependence factor. Since the relative values of the Flory-Huggins parameters vary with a different rate, the separation of different polymer components will occur at different temperatures and a two-step self-assembly will be observed. Figure 2 illustrates this for the choice ${\text{X}}_{AB}$*N* = ${\text{X}}_{BC}$*N* = $\frac{3}{4}{\text{X}}_{AC}$*N*. At high temperatures (low X*N*) no phase separation occurs as all three components are able to mix. Lowering the temperature (increasing X*N*) the A and C components becomes sufficiently immiscible with the B component to induce phase segregation (*TP*_2_ in Figure 2(g)), and the structure shown in Figure 2(d–f) emerges. Upon continued lowering of the temperature, the A and C components also become immiscible and separate through a secondary phase transition (*TP*_1_) to form the structure shown in Figure 2(a–c). This demonstrates that a two-step phase transition can be achieved as a function of temperature variations when the system is slightly asymmetric in its interactions. Again, the free energies of the nearby morphologies are shown to demonstrate that the illustrated morphologies indeed are the minimum energy configurations in the various regions of parameter space [11].

### 3.2. Non-selective Solvent

Experimentally, temperature can be varied to induce ordering and disordering. Alternatively, solvent is often used to dilute the interactions and achieve similar results. For non-selective solvents, this effect is quantified in the familiar dilution approximation [5, 12–14], which states that the first order effect of a solvent is to limit immiscibility according to the relationship

$${\left(\text{X}N\right)}_{\text{eff}}=\varphi \text{X}N.$$

Here we will determine the effect on the ordering mechanisms of the triblock copolymer *without* using the dilution approximation. We fix ${\text{X}}_{AB}$*N* = ${\text{X}}_{BC}$*N* = ${\text{X}}_{AC}$*N* and ${\text{X}}_{AS}$*N* = ${\text{X}}_{BS}$*N* = ${\text{X}}_{CS}$*N* and vary the solvent volume fraction (1 − *φ*). The qualitative behavior of the melt with the solvent is found to be the same for a large range of the parameters describing the solvent-polymer interaction (i.e. ${\text{X}}_{AS}$, ${\text{X}}_{BS}$ and ${\text{X}}_{CS}$).

**Figure 3.**
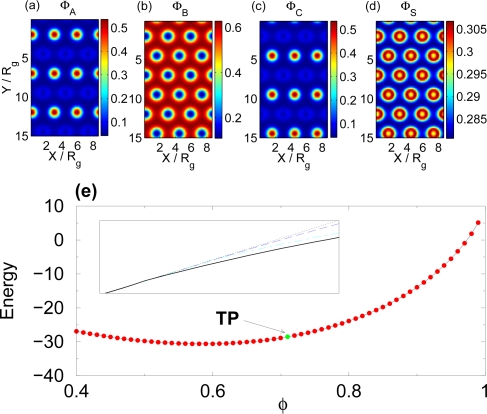
(a), (b), (c) and (d) show the spatial densities of the A, B and C components of the polymer as well as the solvent for ${\text{X}}_{AB}$*N* = ${\text{X}}_{AC}$*N* = ${\text{X}}_{BC}$*N* = 50 and ${\text{X}}_{AS}$*N* = ${\text{X}}_{BS}$*N* = ${\text{X}}_{CS}$*N* = 10, and *φ* = 0.71. (e) Free energy of the mixture as a function of the polymer volume fraction *φ*. The order-disorder transition point (TP) is marked on the figure. The inset shows the free energies of the disordered phase (short-dashed line), the lamellar phase (short-dashed/long-dashed line) and the hexagonal phase in which A and C components mix (long-dashed line) in the neighborhood of the transition point.

Because of the dilution effect of uniformly decreasing interaction parameters, we expect in this case a one-step self-assembly process, as in the case of varying the temperature. The reason is that the interaction between the polymer components is symmetric, and the solvent will interact with the three components with exactly the same strength. In Figure (3a) we show the concentrations of the polymer components and the solvent for solvent volume fraction *φ_S_* = 0.29.

Figure 3 confirms hese expectations. In the absence of solvent (*φ* = 1) all three components segregate into spatially iseparated domains. This scenario persists as small amounts of solvent is added to the mixture. Since the solvent is non-selective it distributes roughly uniformly throughout the system (see Figure 3(d) and note that the variation in solvent concentration is less than 5%) with a small excess (*<* 5 % above average solvent concentration) at the domain interfaces. This excess phenomena is known to be an energetic effect [5]. Once the overall volume faction of the solvent reaches 0.29 (marked as TP in Figure 3(e)), the polymer is rendered so miscible that a single step order-disorder transition occurs.

Similarly, if we choose *A_AB_* = *A_BC_* ≠ *A_AC_* and select any corresponding ${\text{X}}_{AB}$*N*, ${\text{X}}_{BC}$*N* and ${\text{X}}_{AC}$*N* (that is, pick a single temperature point) then upon adding non-selective solvent while holding these parameters fixed, we can induce two-step self-assembly just as if temperature were varied for components with an unequal temperature response. This is again consistent with dilution arguments. Thus either one or two-step self-assembly may be achieved through the use of non-selective solvent rather than through varying temperature.

### 3.3. Selective Solvent

**Figure 4.**
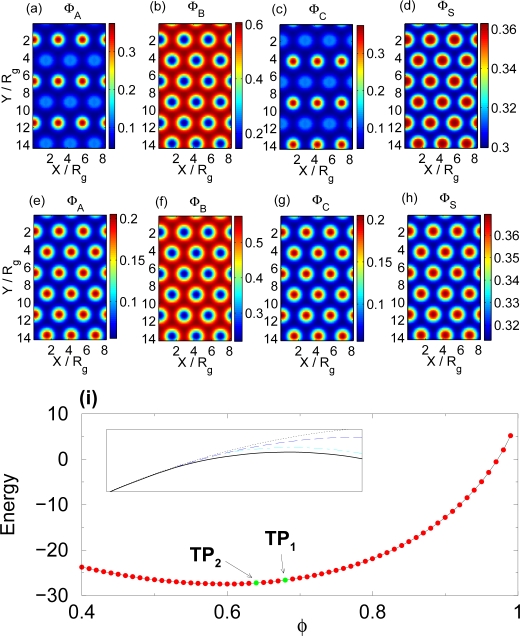
(a), (b), (c) and (d) show spatial monomer densities of the A, B, and C components of the polymer as well as the solvent density for polymer volume fraction *φ* = 0.68, ${\text{X}}_{AB}$*N* = ${\text{X}}_{AC}$*N* = ${\text{X}}_{BC}$*N* = 50, ${\text{X}}_{AS}$*N* = ${\text{X}}_{BS}$*N* = 5 and ${\text{X}}_{CS}$*N* = 30. (e), (f), (g) and (h) show the concentration of the A, B and C components of the polymer as well as the solvent for polymer volume fraction *φ* = 0.67 (the Flory-Huggins parameters are the same). (i) Free energy of the melt (dimensionless units) as a function of the polymer volume fraction *φ*. The transition points (TP_1_) and (TP_2_) are marked on the figure. The inset shows the free energies of the disordered phase (short-dashed line), the lamellar phase (short-dashed/long-dashed line) and the hexagonal phase in which A and C components mix (long-dashed line) in the neighborhood of TP_2_.

The symmetry in the interaction can also be broken by using a selective solvent. The selective solvent will have different concentration in regions occupied by different polymer components. This will affect the interactions between polymer components and break the symmetry. To demonstrate this effect we choose a solvent that is more miscible with the *A* and *C* components of the polymer than it is with the B component. Selecting ${\text{X}}_{AS}$*N* ≃ ${\text{X}}_{BS}$*N* < ${\text{X}}_{CS}$*N*, and keeping ${\text{X}}_{AB}$*N* = ${\text{X}}_{BC}$*N* = ${\text{X}}_{AC}$*N* we expect the presence of the selective solvent to produce a two-step self-assembly upon increasing solvent content. As the selective solvent is added to the polymer melt, it builds up primarily in the A and C rich domains (see Figure 4(a–b)), which in turn means that the solvent will dilute the interactions between A and C monomers until they become miscible. This occurs for *φ* ≃ 0.68 (*TP*_1_ in Figure 4(i)) where the mixture transitions to the structure illustrated in Figure 4(e–h). The addition of slightly more solvent *φ* ≃ 0.64 causes a complete loss of structure as the order-disorder transition occurs (*TP*_2_).

In general, the phase behavior of a triblock melt with solvent is very complicated. The complete phase diagram is embedded into a 6 + 2 dimensional parameter space (6 for the Flory-Huggins parameters plus two for the triblock composition and the volume fraction of polymer). So far we have dealt with simple transitions, which are straightforward extrapolations from a diblock to a triblock copolymer. Using polymers consisting of different components, and different types of solvent (which leads to different Flory-Huggins parameters) one might expect more complicated transitions. In the experiments by Yamauchi *et al.* [2] a two step transition is studied in a triblock copolymer under the influence of solvent but the observed behavior is slightly different from the two-step mechanisms we have described so far. The difference lies in the fact that initially, two of the components of the polymer are mixed, while separated from the third. As the solvent concentration increases, the segregation increases and the A and C components phase separate. In the transitions we have studied earlier, our polymer components were completely separated initially. Due to this initial difference, we term the first step of the transition observed in the experiment “reversed transition”.

Although it is very difficult to explore the full parameter space, we can determine a physical mechanism that explains this reverse transition. We assume that initially the *A* and *C* component of our polymer should be mixed. To achieve miscibility of the *A* and *C* polymer components at low/no solvent density we have to chose a polymer with a weak interaction between these two components therefore we have to reduce the ${\text{X}}_{AC}$*N* parameter. Since we expect these two components to separate by adding solvent, this parameter cannot be much lower than the critical transition value. A selective solvent with a strong attractive interaction with the *A* component will tend to concentrate in the A rich domains. If at the same time this solvent strongly repels the *C* polymer component, it will cause an effective immiscibility of the A and C polymer components.

**Figure 5.**
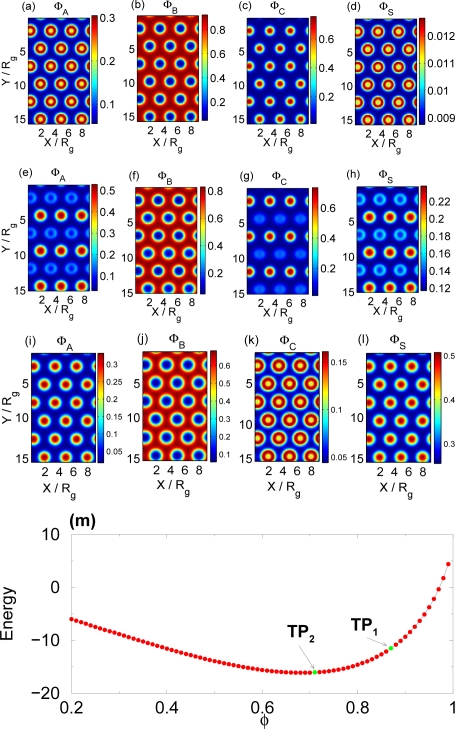
(a), (b) and (c) shows spatial monomer densities of A, B, and C components of the polymer and (d) the density of the solvent for polymer volume fraction *φ* = 0.99, ${\text{X}}_{AB}$*N* = 34, ${\text{X}}_{AC}$*N* = 24, ${\text{X}}_{BC}$*N* = 50 and ${\text{X}}_{AS}$*N* = −10, ${\text{X}}_{BS}$*N* = ${\text{X}}_{CS}$*N* = 90. (e), (f) and (g) shows spatial monomer densities of the A, B, and C components of the polymer and (h) the density of the solvent for polymer volume fraction *φ* = 0.886. (Flory-Huggins parameters are as in (a–d)). (i), (j) and (k) shows spatial monomer densities of the A, B, and C components of the polymer and (l) the density of the solvent for polymer volume fraction *φ =* 0.65. (m) Free energy of the mixture as a function of the polymer volume fraction *φ*. The transition points (TP _1_), (TP_2_) are marked on the figure.

To demonstrate this we choose ${\text{X}}_{AC}$*N* ≲ ${\text{X}}_{AB}$*N* ≲ ${\text{X}}_{BC}$*N* with ${\text{X}}_{AC}$*N* less than but very close to the order-disorder transition between *A* and *C,* and take *φ* = 1. A semi-ordered structure, results (see Figure 5(a–d)) where *A* and *C* are mixed in domains with almost no content of *B*. It is worth noting that although *A* and *C* are mixed, *C* predominately forms the core of the domains in order to minimize the highly unfavorable *B-C* interactions. We choose a strongly selective solvent with ${\text{X}}_{AS}$*N* < 0 while ${\text{X}}_{BS}$*N* = ${\text{X}}_{CS}$*N* are large. Also, the negative ${\text{X}}_{AS}$*N* value means that the *A* species lowers the free energy by residing in an environment of solvent compared to an A rich environment. Upon increasing the solvent content (beyond *TP*_1_ in Figure 5(m)), the system becomes fully ordered – *A, B* and *C* all segregate in spatially separate domains as shown in Fig. 5(e–h). This is analogous to the “reversed transition” observed in the experiment; usually the solvent increases the disorder since it dilutes the segregation and promotes mixing. In this case, the *negative* segregation ${\text{X}}_{AS}$*N* promotes aggregation and thus order.

Further increasing the solvent content (beyond *TP*_2_) induces re-entrant behavior in that the system becomes semi-ordered once again – *A* and *C* mix again as illustrated in Figure 5(i–l). Note that the *A* and *C* internal morphologies have reversed their patterns so that *A* now forms the cores in the mixed domains; this is similar to the reversal of morphologies seen in diblock solutions with strongly selective solvents [14]. The re-entrance occurs as a result of the *B-C* interaction becoming increasingly diluted whereas both *B* and *C* interaction with the solvent remains very unfavorable. It therefore becomes attractive for the system to use the *C* monomers’ weak interactions with the *A* monomers to minimize the interaction with the solvent.

As shown in Figure (5m), the free energy curve indicates no macrophase separation over this range of solvent volume fractions. This diagram also summarizes the multiple assembly steps in this selective solvent-triblock system, as a function of solvent volume fraction.

## 4. Conclusions

We have illustrated ordering mechanisms in a triblock copolymer system as they appear as a function temperature and (non-selective and selective) solvent content. For temperature dependent X*N* values in a pure (no solvent) triblock melt, we find that one or two-step ordering is possible, the former occurring when ${\text{X}}_{AB}$*N* = ${\text{X}}_{BC}$*N* = ${\text{X}}_{AC}$*N* with all segregation being varied with temperature equally, and the latter occurring when ${\text{X}}_{AC}$*N* is lower than ${\text{X}}_{AB}$*N* or ${\text{X}}_{BC}$*N*.

The addition of non-selective solvent may also induce either one or two-step ordering processes depending on whether the X*N* values of *A, B* and *C* triblock constituents are held fixed at equal, or unequal values. For ${\text{X}}_{AB}$*N* = ${\text{X}}_{BC}$*N* = ${\text{X}}_{AC}$*N*, one-step ordering occurred, but for ${\text{X}}_{AB}$*N* = ${\text{X}}_{BC}$*N* *≠* ${\text{X}}_{AC}$*N*, two-step ordering resulted.

Adding selective solvent causes two-step self-assembly when the solvent is sufficiently selective, and for certain choices of system parameters, a much more complicated route of self-assembly was found.

## References

[1] Yamauchi K, Hasegawa H, Hashimoto T, Köhler N, Knoll K (2002). Synthesis and morphological studies of polyisoprene-block-polystyrene-block-poly(vinyl methyl ether) triblock terpolymer. Polymer.

[2] Yamauchi K, Hasegawa H, Hashimoto T, Nagao M (2003). Complex microphase separation and microdomain structures in poly(isoprene)-block-poly(D-8-styrene)-block-poly(vinyl methyl ether) triblock terpolymer. J. Appl. Cryst.

[3] Maniadis P Thompson RB Rasmussen KØ Lookman T Ordering mechanisms in triblock copolymers Phys Rev E 2004 69, 031801/1-6. 10.1103/PhysRevE.69.031801 15089313

[4] Vavasour JD, Whitmore MD (1993). Self-consistent field theory of block copolymers with conformational asymmetry. Macromolecules.

[5] Naughton JR, Matsen MW (2002). Limitations of the dilution approximation for concentrated block copolymer/solvent mixtures. Macromolecules.

[6] Tzeremes G, Rasmussen KØ, Lookman T, Saxena A (2002). Efficient computation of the structural phase behavior of block copolymers. Phys Rev E.

[7] Rasmussen KØ, Kalosakas G (2002). Improved numerical algorithm for exploring block copolymer mesophases. J. Polymer Sci. Part B: Polymer Phys.

[8] Doi M (1996). Introduction to Polymer Physics.

[9] Mai S-M, Fairclough JPA, Terrill NJ, Turner SC, Hamley IW, Matsen MW, Ryan AJ, Booth C (1998). Microphase separation in poly(oxyethylene)-poly(oxybutylene) diblock copolymers. Macromolecules.

[10] Mai S-M, Mingvanish W, Turner SC, Chaibundit C, Fairclough JPA, Heatley F, Matsen MW, Ryan AJ, Booth C (2000). Microphase-separation behavior of triblock copolymer melts. Comparison with diblock copolymer melts. Macromolecules.

[11] It is important to note that all our simulations are performed in two-dimensions, which excludes certain morphologies than can only be realized in three-dimensions. It is therefore possible that, as large amounts of solvent is added, we may be missing low energy, high-curvature morphologies that are only realizable in three-dimensions. However, this does not change the phenomenology this paper describes.

[12] Whitmore MD, Noolandi J (1990). Self-consistent theory of block copolymer blends: neutral solvent. J. Chem. Phys.

[13] Whitmore MD, Vavasour JD (1992). Self-consistent mean field-theory of the microphase diagram of block copolymer neutral blends. Macromolecules.

[14] Huang C-I, Lodge TP (1998). Self-consistent calculations of block copolymer solution phase behavior. Macromolecules.

